# circRNAs shed light on cancer diagnosis and treatment

**DOI:** 10.1186/s12943-022-01580-2

**Published:** 2022-04-29

**Authors:** Christophe Nicot

**Affiliations:** Department of Pathology and Laboratory Medicine, University of Kansas Medical Center, 3901 Rainbow Boulevard, Kansas City, KS 66160 USA

Circular RNAs (circRNAs) are a subgroup of single-stranded endogenous RNAs which exert differential expression pattern between normal and cancerous tissues and function as important regulators in cancer initiation and progression. However, comprehensive characterization of circRNA landscape across cancer types is still lacking. In a recent article published in *Molecular Cancer*, Wang and his colleagues have now placed seven types of tumors in a unified analytic framework [1], all with matched tumors and corresponding normal tissues. This work illustrated the detailed picture of circRNA expression signatures among solid tumors and highlighted the significance of these dysregulated circRNAs in cancer pathogenesis as well as their utility as potential indicators or therapeutic agents.

Through rRNA depleted transcriptome sequencing, the authors identified a total of 59,056 circRNAs, the majority of which were lowly expressed, while a subset of circRNAs exhibited much higher abundance than their cognate linear transcripts, indicating their biological significance in homeostasis. Using stringent criteria, the authors pictured the distinct circRNA expression signatures among seven types of solid tumors. The dysregulated circRNAs exhibited cancer-specific expression or shared common expression pattern across cancers, implying their diverse functions in cancer progression and their diagnostic potential in multiple cancers. Among the aberrant circRNAs, circLIFR showed an overall downregulation in tumors. Significantly, circLIFR was experimentally validated as a bona fide circRNA which inhibited tumor metastasis in vitro and in vivo*,* demonstrating circLIFR may serve as a therapeutical target in metastatic cancer. Consistently, the RNA-seq results suggested the ability of circLIFR to alter the expression pattern of some metastasis-related genes involved in cell adhesion and epithelial-mesenchymal transition (EMT). Collectively, this study by Wang et al. certainly illustrates the comprehensive circRNA profiles in multiple solid tumors and highlights the potential of circRNAs as important diagnostic tools or therapeutic targets.
